# Optimization of the gantry drive through dynamic testing and comparison with the crankset in a pilot study

**DOI:** 10.1038/s41598-025-00397-5

**Published:** 2025-05-07

**Authors:** Łukasz Bereś, Justyna Pyrzanowska, Dagmara Mirowska-Guzel, Marcin Obszański, Paweł Pyrzanowski

**Affiliations:** 1 Institute of Aeronautics and Applied Mechanics, Warsaw University of Technology, Nowowiejska str. 24, Warsaw, 00-665 Poland; 2 Medical University of Warsaw, Żwirki i Wigury str. 61, Warsaw, 02-091 Poland

**Keywords:** Urban ecology, Civil engineering, Mechanical engineering, Engineering

## Abstract

The aim of the research was to conduct a pilot optimization of the gantry drive with a swinging pressure plate, which resembles the leg press exercise, using dynamic tests and to gather users’ opinions about this new drive. Moreover, the efficiency of the gantry drive was compared with the commonly known crankset. The research was conducted at a load of 50 W on a group of young men, and the results were subjected to statistical analysis. The research has shown that the gantry drive with guides mounted at a positive angle provides the highest efficiency, and users evaluated this mechanism positively despite their first encounter with it. Additionally, it has been shown that in terms of efficiency, when operating at low power, the gantry drive is generally similar to the crankset. The gantry drive takes up less volume than the crankset, making it well-suited for compact road vehicles and advantageous for water vehicles as well.

## Introduction

The gantry drive was discovered in 1948 in England^1^. A review of the literature shows that it has been forgotten for unknown reasons. The gantry drive was rediscovered in 2019 in Poland^2^. A number of tests and static analyses were carried out^3,4^, which allowed the discovery of several improvements to the original concept of this drive^5–7^.

Nominally, the gantry drive consists of a pressure plate mounted on a linear trolley that is kinematically coupled to the vehicle’s drive wheel. There is a one-way clutch in the drive system that allows the gantry to retract when the vehicle is moving forward. The pressure plate is pressed with both legs by a person who puts his back against the back of the seat.

**Fig. 1:**
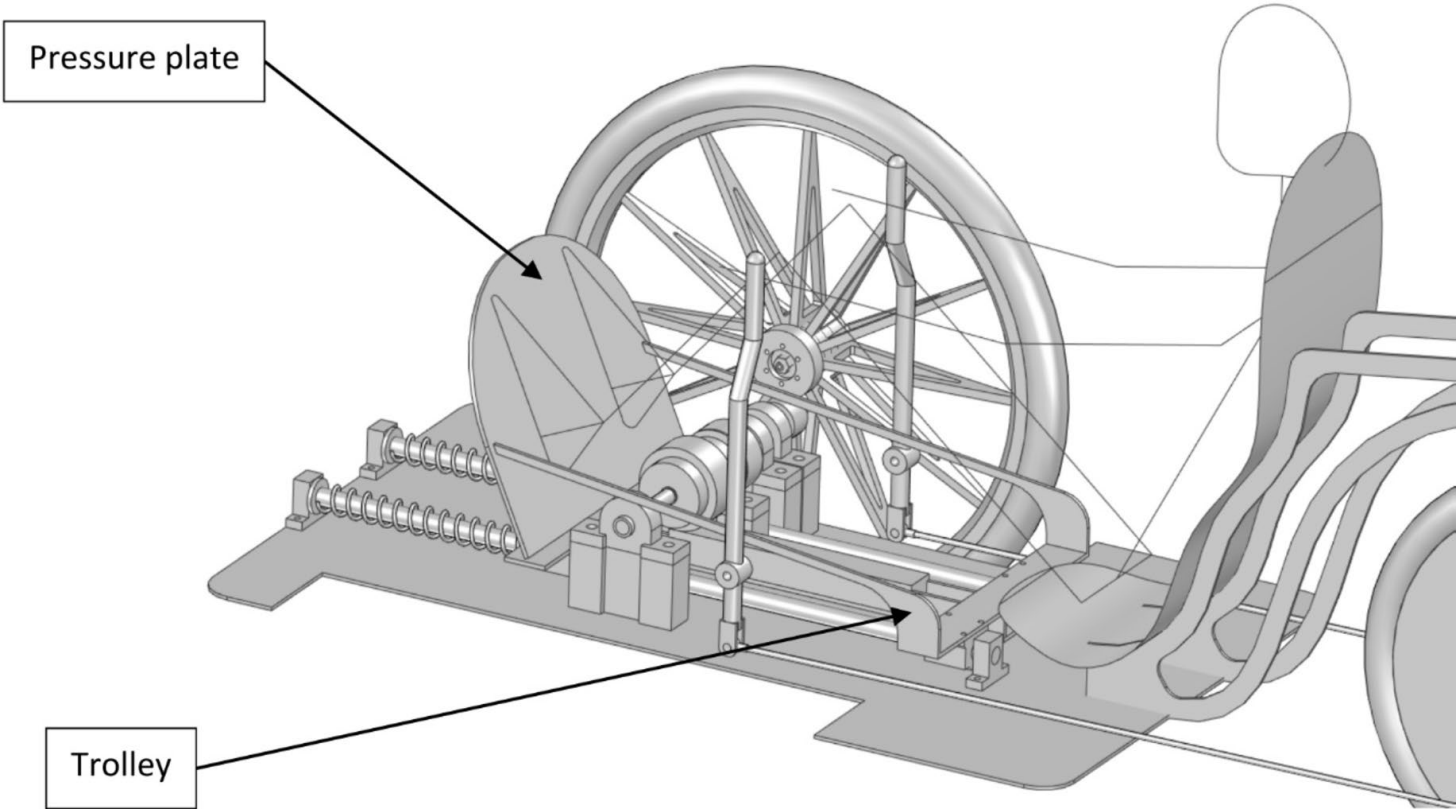
Basic version of the gantry drive.

**Fig. 2:**
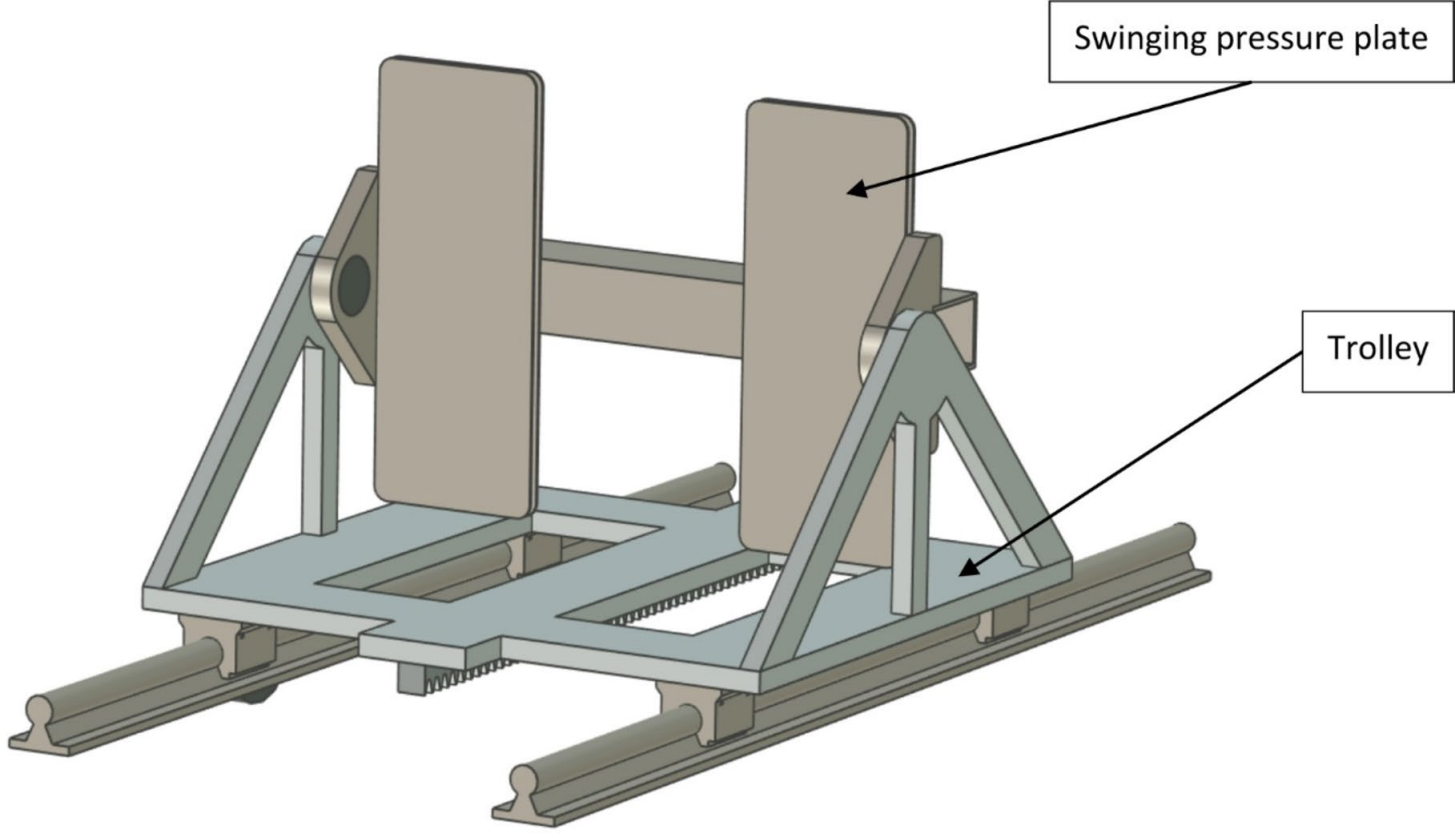
Improved gantry drive equipped with a swinging pressure plate.

In the original solution, the pressure plate is mounted stationary on a trolley (see Fig. 1). One of the key improvements of this drive is the mounting of the pressure plate in a swinging manner relative to the trolley (see Fig. 2). This solution allows the gantry to increase its stroke and reduce the load on the human musculoskeletal system. The gantry drive with a swinging pressure plate was the subject of the research.

The aim of the research was to optimize the angular position of the gantry drive guides using dynamic tests. In addition, it was planned to check what people’s feelings are about this new gantry drive. The aim of the research was also to check how the gantry drive compares to the crankset in terms of efficiency calculated on the basis of air flow.

This article is essentially a continuation of research on drives for ultra-light personal vehicles^8^ equipped with crankset. The research was conducted in a similar way to the research on crankset, in order to be able to easily compare both techniques for receiving mechanical energy from humans. The research was carried out on the same group of people and with a time difference of approximately one month. The presented tests are the first known dynamic tests ever carried out on the gantry drive.

**Fig. 3:**
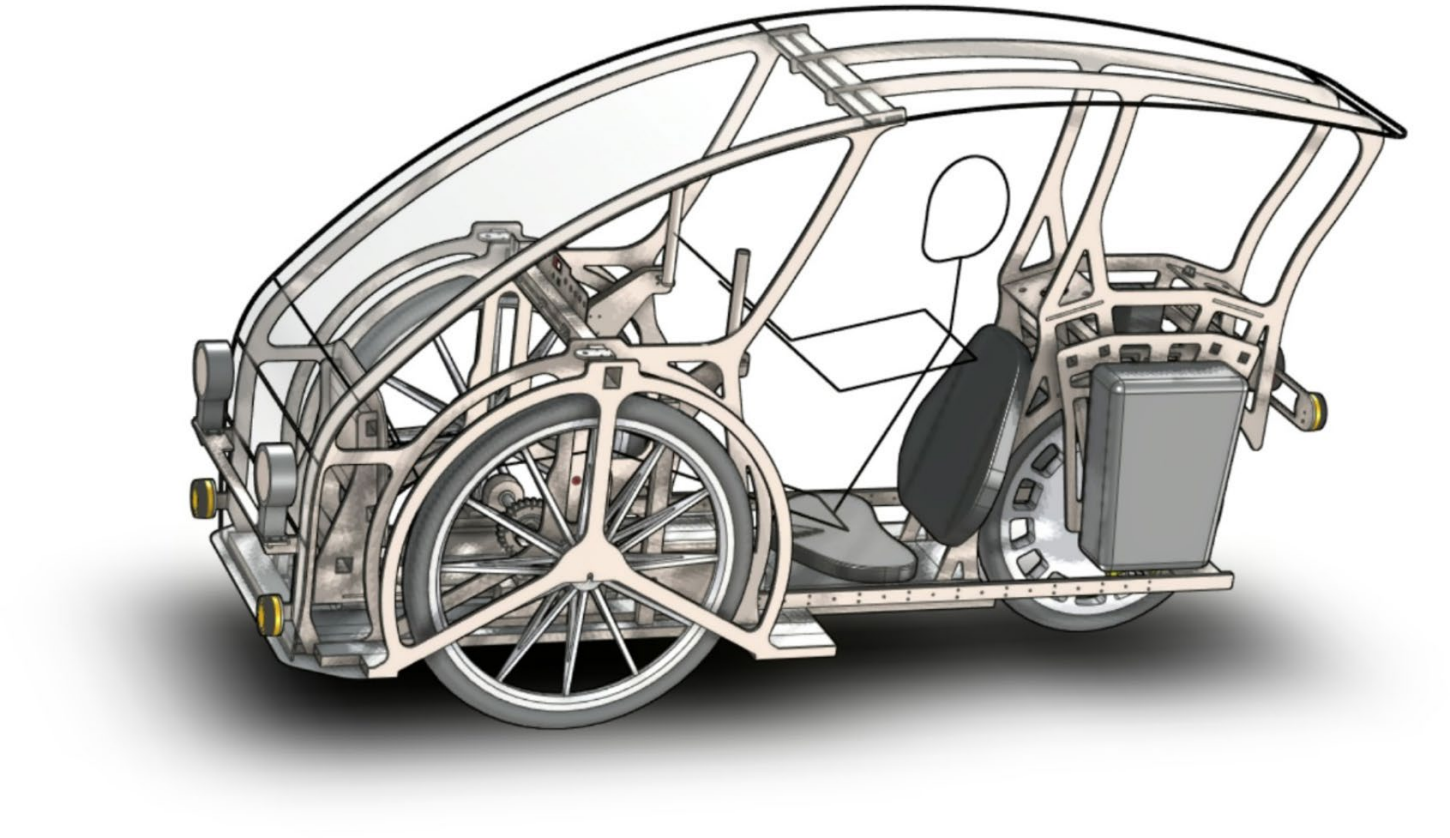
The outline of a three-wheeled personal vehicle equipped with a gantry drive^9^.

**Fig. 4:**
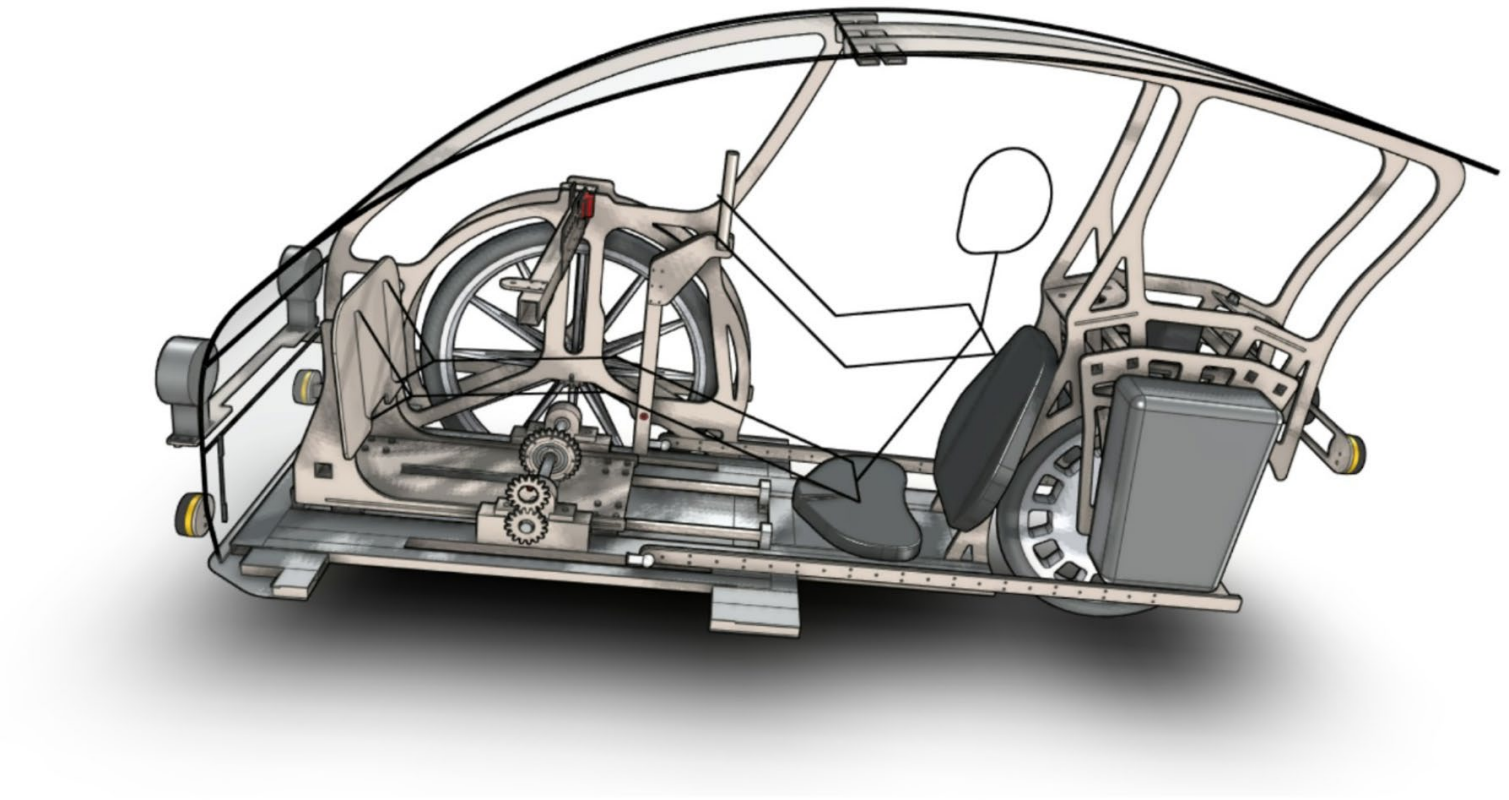
The interior of a three-wheeled personal vehicle equipped with a gantry drive^9^.

The concept of personal vehicles was developed as a response to people’s problems related to broadly understood passenger transport. Personal vehicles are bicycles with the functional characteristics of a passenger car. An example of a personal vehicle without side covers is shown in Fig. 3. The interior of a personal vehicle equipped with a gantry drive is shown in Fig. 4. Personal vehicles supported by an electric motor are a solution that can solve many problems of modern civilization.

In recent years, the topic of ecology and sustainable development has appeared particularly frequently. It is predicted that the cities of the future will become green places^10,11^. Personal vehicles allow users to directly reach places outside the main public transport lines, which is expected to lead to a more even density of people in the available city space, without the accumulation of people at the main transport hubs. The main life now takes place in places where communication is paradoxically good, which in turn leads to many inefficiencies, including: creating congestion, higher accident rates, inefficient use of urban land, environmental pollution, adverse economic impacts, urban sprawl and ultimately degrades the overall quality of people’s lives^12^. Another extremely important factor that worsens people’s lives is noise pollution in cities^13–15^, which can be limited by personal vehicles. Personal vehicles fit well into the concept of sustainable cities of the future and constitute an alternative to the commonly known passenger cars and public transport, it seems that this may be a solution to the existing, complex problems of cities.

People from small towns and villages, in turn, have been struggling especially in recent years with the problems of social exclusion^16–22^, which is mainly caused by the depopulation of these areas, which causes inefficiency^23,24^. This problem particularly affects young and older people and affects the entire world. A great chance to solve this problem is seen in micro vehicles, which would be something between a passenger car and a bicycle^25^. Cars have a relatively high cost to earnings in small towns, while people often do not use bicycles due to fear of bad weather, reported lack of stability of the vehicle and fear of traveling on roads where cars travel^26^. Public transport is also often rejected due to uncertainty^27^, which leads to a further reduction in the frequency of public transport. A personal vehicle may be an economically effective solution, especially since it will be a bicycle from a legal point of view.

The concept of light three-wheeled and four-wheeled vehicles was intensively developed for a certain period in the history of the automotive industry^28^. Observing the evolution of the car, it seems that the need to develop increasingly higher speeds led to the creation of modern cars according to the mechanism presented below. To develop higher speeds, greater engine power is needed, which leads to an increase in the engine mass and then to an increase in the mass of the vehicle structure in order to be able to transfer dynamic loads. In the initial phase of motorization, combustion engines had quite a large mass, so they could not be used in vehicles based on bicycles. A similar situation occurred with electric motors, which, despite their relatively low weight, required heavy batteries. Contemporary existing electric motors with high-power density batteries and, alternatively, modern low-capacity internal combustion engines allow the use of old concepts of light vehicles in a new version.

Personal gantry-powered vehicles are a pro-ecological solution, forcing human movement and thus improving health, which can lead to a more sustainable, dispersed development of people, which ultimately seems to improve the quality of life of all people both in cities, small towns and villages.

**Fig. 5:**
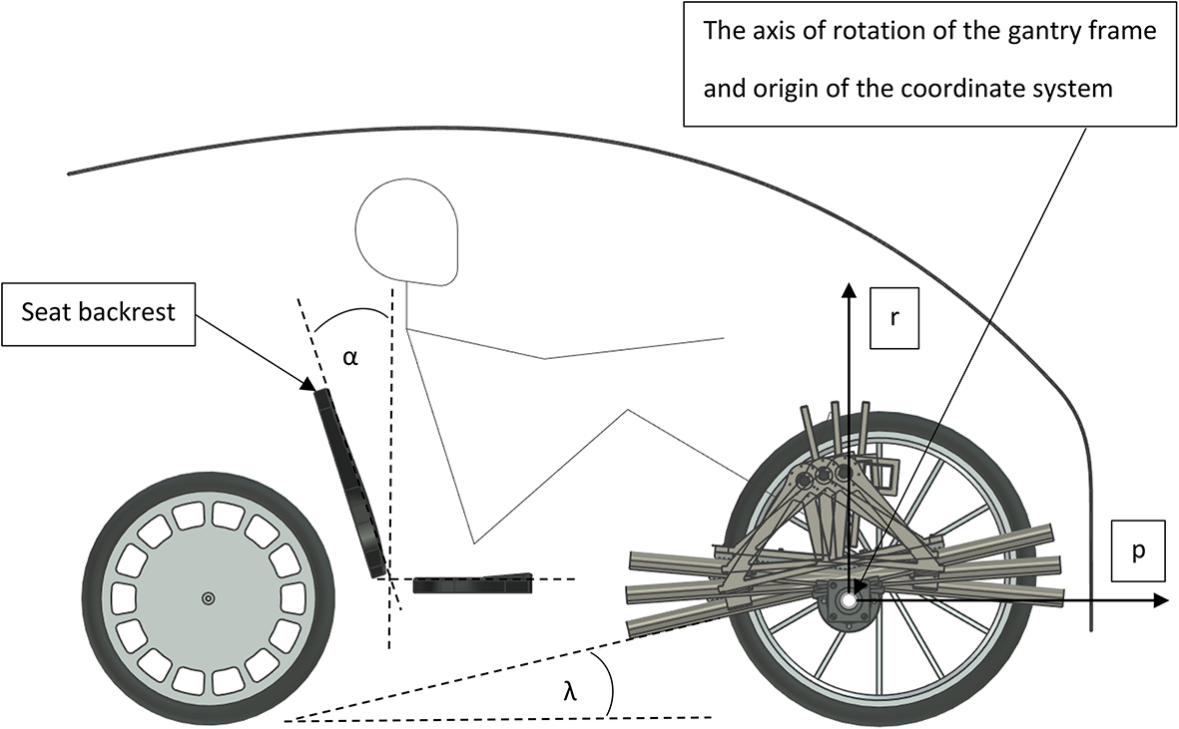
Parameter (λ) that were considered during the tests.

Figure 5 shows the gantry drive with a swinging pressure plate installed in a three-wheeled personal vehicle. The position of the seat relative to the origin of the coordinate system was defined on the basis of previously performed static tests to ensure force maximization, which is described in detail in the next chapter. In turn, the seat backrest inclination was chosen to maximize seat ergonomics^8^. There is a person of average height in the vehicle, sitting in a seat which is mounted at such a distance from the gantry drive to enable symmetrical operation of the trolley in relation to the trolley’s linear guides. The aim of the research was to check what angular position λ of the linear guides would be the most favourable, which is the main subject of the research.

## Materials and methods

The research used a dynamic test stand used in previous analogous studies aimed at optimizing the position of the seat in relation to the crankset^8^. All research methods were the same in order to be able to compare the gantry drive with crankset as accurately as possible.

The research carried out can be divided into two parts. The first part is dynamic research on a group of people. The second part is statistical analysis, aimed at selecting the best angular position of the gantry drive guides and comparing the gantry drive with crankset.

### Dynamic research (determination of efficiency and assessment of ergonomic aspects)

The test stand consisted of a frame, a seat and the gantry drive. A magnetic brake was connected to the test stand, which controlled the human load, expressed in watts, based on information about the human’s age, height, and weight. The jacks controlling the seat’s vertical position were locked in a defined position. The angular position of the linear guides of the gantry drive was changed using a jack. A linear potentiometer was used to measure the position of the seat in the horizontal direction. A torque sensor and a rotary encoder were used to measure the energy received from the human. An air flow sensor was used to measure the energy expended by a person; energy was determined on the basis of the air flow. The magnetic brake worked independently of this measurement. There was a monitor in front of the test station to display instructions for the test person to maintain the correct course of the test procedure. The test subject had an ECG sensor connected to monitor the heart rate.

The key equipment in the measurement chain^8^ was characterized by the following measurement errors:

- National Instruments measuring card (USB-6251): residual gain error (ppm of reading) was 60, residual offset error (ppm of range) was 20,
- Rotary Encoder (PIB406C-3600-G5-24-C, 3600 imp/rev): accuracy up to ± 0.5°,
- Torque Sensor by NCTE AG (DFM22-250-S round shaft, 250 Nm): hysteresis and linearity error: <1%FS, repeatability: ±0.1%FS,
- turbine from MWE-1 energy expenditure meter: measuring error of the air flow: ± 5%,
- Local seat position: potentiometer repeatability < 1%, total position error < 10 mm (the structure of the position was rigid, but had its flexibility).

**Fig. 6:**
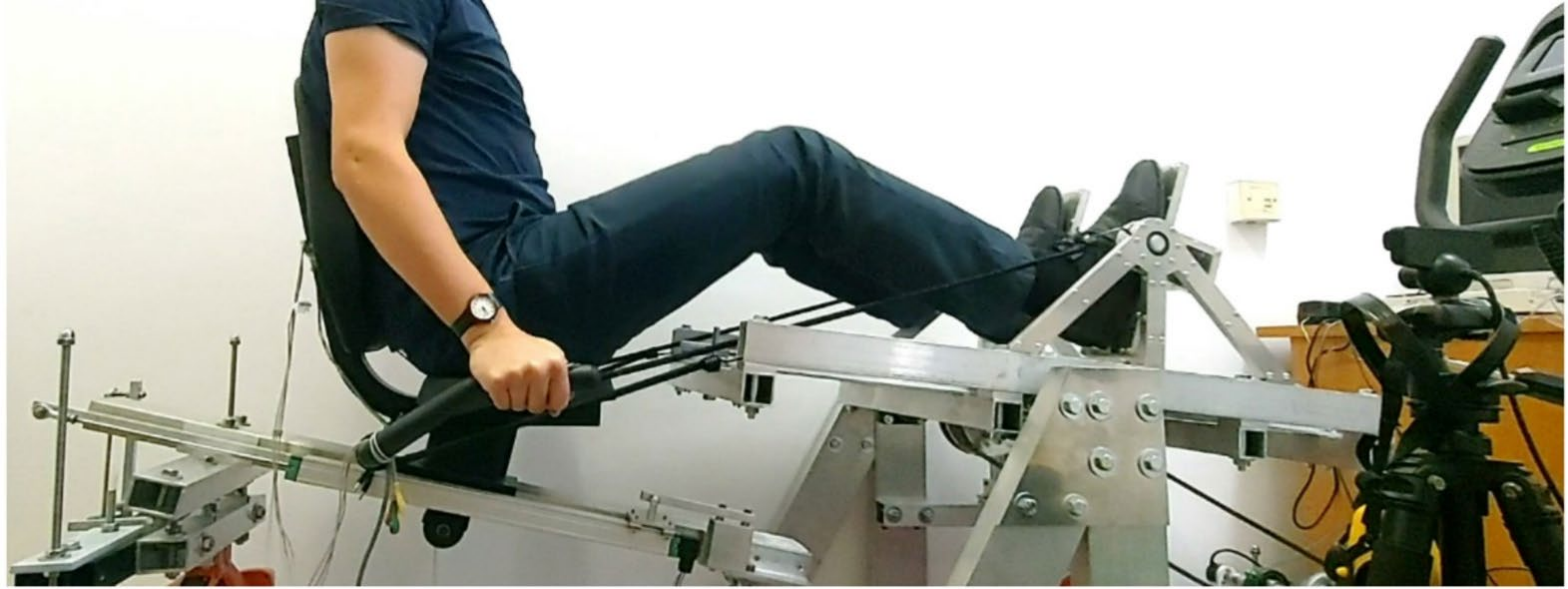
An example of a person on a test stand.

Figure 6 shows a test stand with a person visible during the demonstration of the test. The participants have given informed consent for the publication of the image in an open access online publication.

**Fig. 7:**
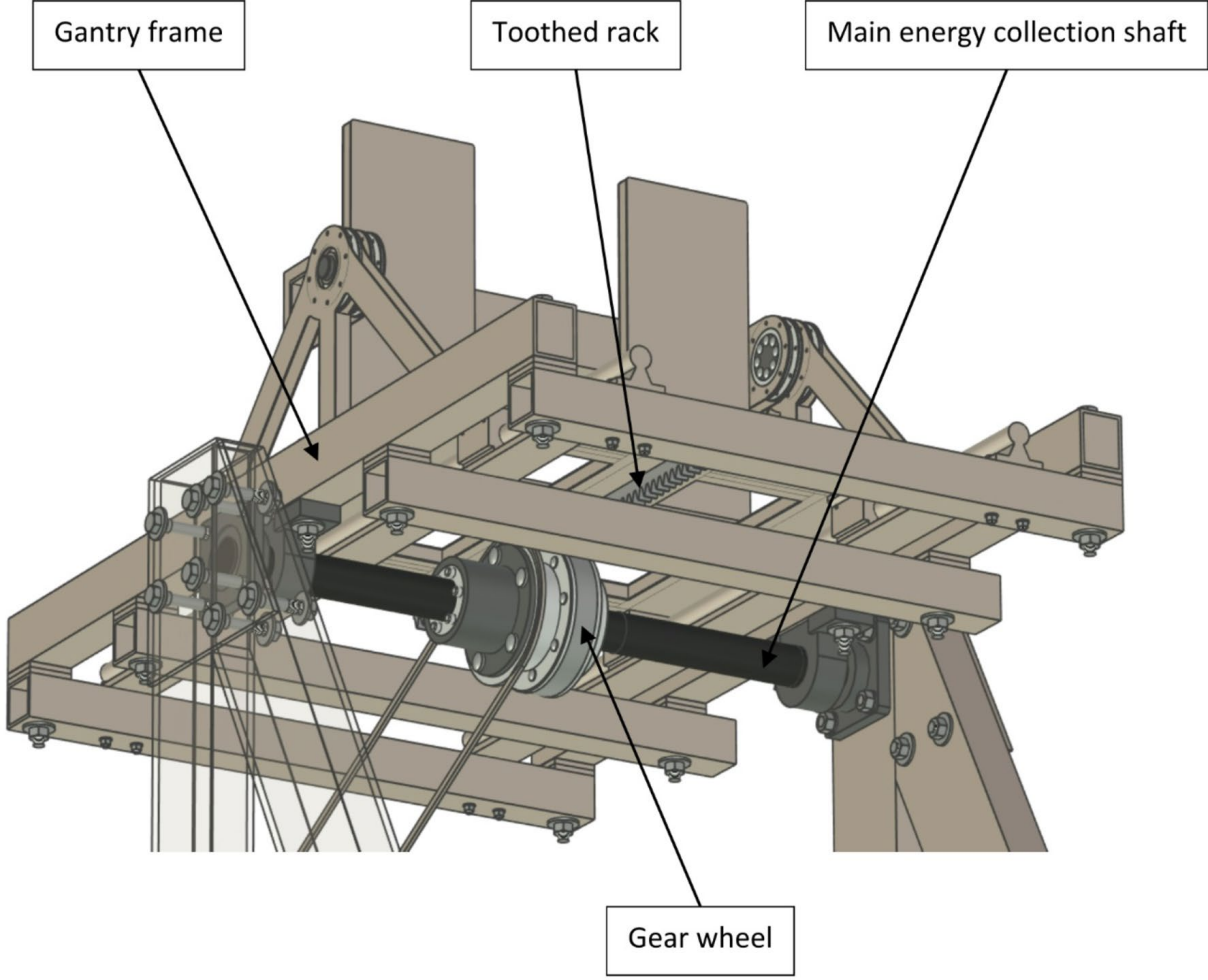
Test stand design seen from below.

In order to simplify the construction of the test stand, the frame of the entire gantry was suspended on the main energy collection shaft (see Fig. 7). A one-way clutch was installed inside the gear wheel connected to the toothed rack, which enabled the gantry to be retracted. Engaging the gantry drive frame had a negative impact on the movement resistance of the entire drive transmission system, but such a solution allowed the entire gantry frame to rotate without any complications with the meshing between the toothed rack and the gear wheel receiving the energy.

To assess ergonomic aspects, a survey was used in which the study participants were asked to assess which angular position of the guides seemed the most comfortable to use.

### Participants

In the dynamic research took part healthy male students of the same fitness status from Warsaw University of Technology. A total of 36 trials were performed, a number based on the pilot research carried out before the main investigation. The same group of people participated in the gantry drive test as in the case of crankset. The exclusion criteria implemented were the same as in the previous experiment^8^. The same number of tests were planned as in the previous tests on crankset, in order to be able to easily compare both mechanisms. The research group was the same, and the interval between the crankset and the gantry drive tests was approximately one month.

The Bioethics Committee of the Medical University of Warsaw approved all the procedures for the study (decision No. KB/53/2022 of April 11, 2022). All procedures were conducted in accordance with relevant guidelines and regulations. All participants were informed about the purpose of the study during the recruitment process and signed informed consent before participating in the experimental protocol. Immediately before the testing the participants were subject to medical qualification. The tests were carried out at a load of 50 W, therefore no special medical protection was required during the trial. Participants’ data were anonymized and their dissemination was restricted.

### Experimental procedure

Detailed information regarding the position of the seat and the angular position of the linear guides considered during the test are presented below.

The dynamic tests carried out were preceded by static tests, which were aimed at a rough analysis of the gantry drive^3^. During static tests, a surface of maximum forces^4^ was developed to check the trajectory along which the test person’s foot should move in order to ensure the longest possible stay in the area that guarantees the maximization of the pressure force, which should translate into maximization of the power that a person is able to generate. The seat was mounted in relation to the gantry drive in such a way that the human feet followed the maximum force curve as closely as possible. The position is defined for a person of average height, for the gantry drive whose linear guides are in a horizontal position. When designing the angular position λ of the linear guides, as mentioned, the curve of maximum forces was taken into account, as well as the occurrence of the force withdrawing the trolley from the component of the trolley’s gravity force and the space occupied by the human-mechanism system. The study was limited to only 3 positions of the linear guides, but it should be borne in mind that there may be more favourable positions of the guides. Moreover, only rectilinear guides were tested, but there are indications^4^ that curvilinear guides may be a better solution.

The seat was mounted on a guide that was inclined to the horizontal plane by β = 15°. It seems that a better solution for this study would have been to mount this seat guide horizontally, but this would have required a thorough reworking of the test stand, so this was abandoned.

The inclination of the seat backrest, as before, was α = 17.5°. This is a standard, comfortable position that is commonly used in ergometers and is justified by numerous studies^8^.

The position of the gantry frame rotation axis and the energy receiving gear wheel was identical, so it was possible to rotate the gantry frame.

The distance of the pressure plate surface to its rotation axis was e = 0.026 m. In turn, the distance of the pressure plate rotation axis to the rotation axis of the energy receiving wheel and the gantry frame rotation axis, in the direction perpendicular to the linear guides, was f = 0.261 m.

The starting torque for the entire measurement path was 1.2 Nm.

**Fig. 8:**
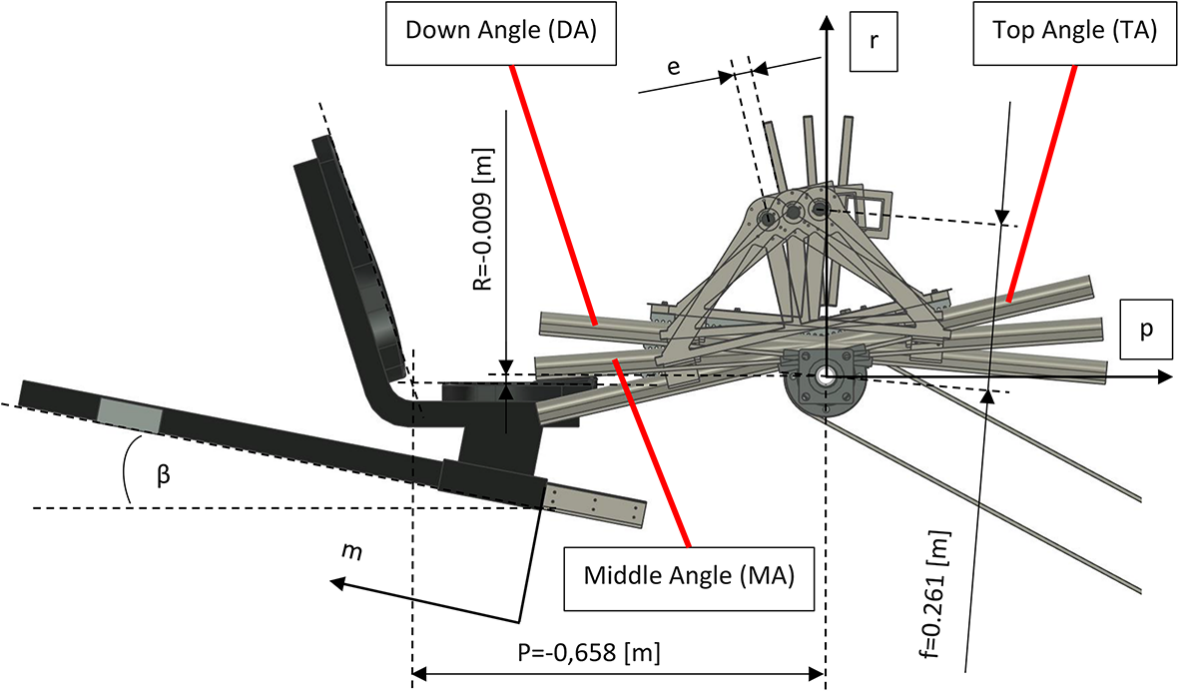
Position of the seat relative to the gantry and various scheduled gantry frame test positions (assuming that m = 0 [m]).

During the study, three angular positions of the linear guides were considered (see Fig. 8): +13° – Top Angle (TA), + 4° – Middle Angle (MA) and − 5° – Down Angle (DA). The warm-up was conducted when the guides were horizontal, i.e. 0° – Zero Angle (ZA).

The examined people were divided into 6 groups. After 6 tests the test sequence was repeated. The detailed test plan is presented in Table 1. The tests were carried out at a load of 50 W.

**Table 1:** Data about the test sequence, the angle position of the linear guides during the test and user preference.

Test number	Test sequence			Seat position m [m]	User preference (best to worst)
	1 (P10)	2 (P12)	3 (P14)		
1	MA	TA	DA	0.310	MA, TA, DA
2	MA	DA	TA	0.340	MA, DA, TA
3	TA	MA	DA	0.267	TA, MA, DA
4	TA	DA	MA	0.325	DA, MA, TA
5	DA	TA	MA	0.305	MA, DA, TA
6	DA	MA	TA	0.298	MA, DA, TA
7	MA	TA	DA	0.400	TA, DA, MA
8	MA	DA	TA	0.372	TA, DA, MA
9	TA	MA	DA	0.292	TA, MA, DA
10	TA	DA	MA	0.212	MA, TA, DA
11	DA	TA	MA	0.249	MA, DA, TA
12	DA	MA	TA	0.320	DA, TA, MA
13	MA	TA	DA	0.269	TA, MA, DA
14	MA	DA	TA	0.321	TA, DA, MA
15	TA	MA	DA	0.338	DA, MA, TA
16	TA	DA	MA	0.268	MA, DA, TA
17	DA	TA	MA	0.292	DA, MA, TA
18	DA	MA	TA	0.277	MA, DA, TA
19	MA	TA	DA	0.362	MA, TA, DA
20	MA	DA	TA	0.397	MA, TA, DA
21	TA	MA	DA	0.329	DA, MA, TA
22	TA	DA	MA	0.293	MA, DA, TA
23	DA	TA	MA	0.334	TA, MA, DA
24	DA	MA	TA	0.361	MA, DA, TA
25	MA	TA	DA	0.276	MA, TA, DA
26	MA	DA	TA	0.322	TA, MA, DA
27	TA	MA	DA	0.272	TA, MA, DA
28	TA	DA	MA	0.361	TA, MA, DA
29	DA	TA	MA	0.317	DA, TA, MA
30	DA	MA	TA	0.382	TA, MA, DA
31	MA	TA	DA	0.275	DA, MA, TA
32	MA	DA	TA	0.326	MA, TA, DA
33	TA	MA	DA	0.246	MA, TA, DA
34	TA	DA	MA	0.382	TA, MA, DA
35	DA	TA	MA	0.303	MA, DA, TA
36	DA	MA	TA	0.281	TA, DA, MA

Detailed research procedure for dynamic tests:

P1. Conducting an interview by the doctor with the tested person regarding e.g. occurrence of diseases that may be contraindications to exercise. Assigning a working number to the tested person. Collecting data on the age, height and weight of the tested person.

P2. Adjusting the gantry frame to the zero angle position (ZA).

P3. Entering the data about the tested person into the computer controlling the magnetic brake and switching on the mode of maintaining the set power of 30 W.

P4. The tested person sits on the seat and sets the distance m of the seat from the gantry, ensuring that the trolley operates in the central part of the gantry frame.

P5. Measurement of pulse and blood pressure using an external measuring device.

P6. Connecting the pulse sensor to the tested person and putting on a half mask equipped with a flow sensor.

P7. Adaptation of the subject’s breathing for 3 min.

P8. Warm-up conducted at the power of 30 W for 1 min and at the same time the test person’s adaptation to the test stand.

P9. A break of 3 min for the tested person. Increasing the load on the magnetic brake to 50 W. Setting the gantry frame to the position in accordance with the planned sequence (TA or MA or DA – see Table 1).

P10. Trial 1 performed by the tested person for 4 min.

P11. A break of 3 min for the tested person. Setting the gantry frame to the position in accordance with the planned sequence (TA or MA or DA – see Table 1).

P12. Trial 2 performed by the tested person for 4 min.

P13. A break of 3 min for the tested person. Setting the gantry frame to the position in accordance with the planned sequence (TA or MA or DA – see Table 1).

P14. Trial 3 performed by the tested person for 4 min.

P15. Measurement of pulse and blood pressure using an external measuring device.

P16. Disconnecting the measuring apparatus from the tested person.

P17. Completion of the questionnaire by the tested person regarding the preferred position of the gantry frame.

**Fig. 9:**
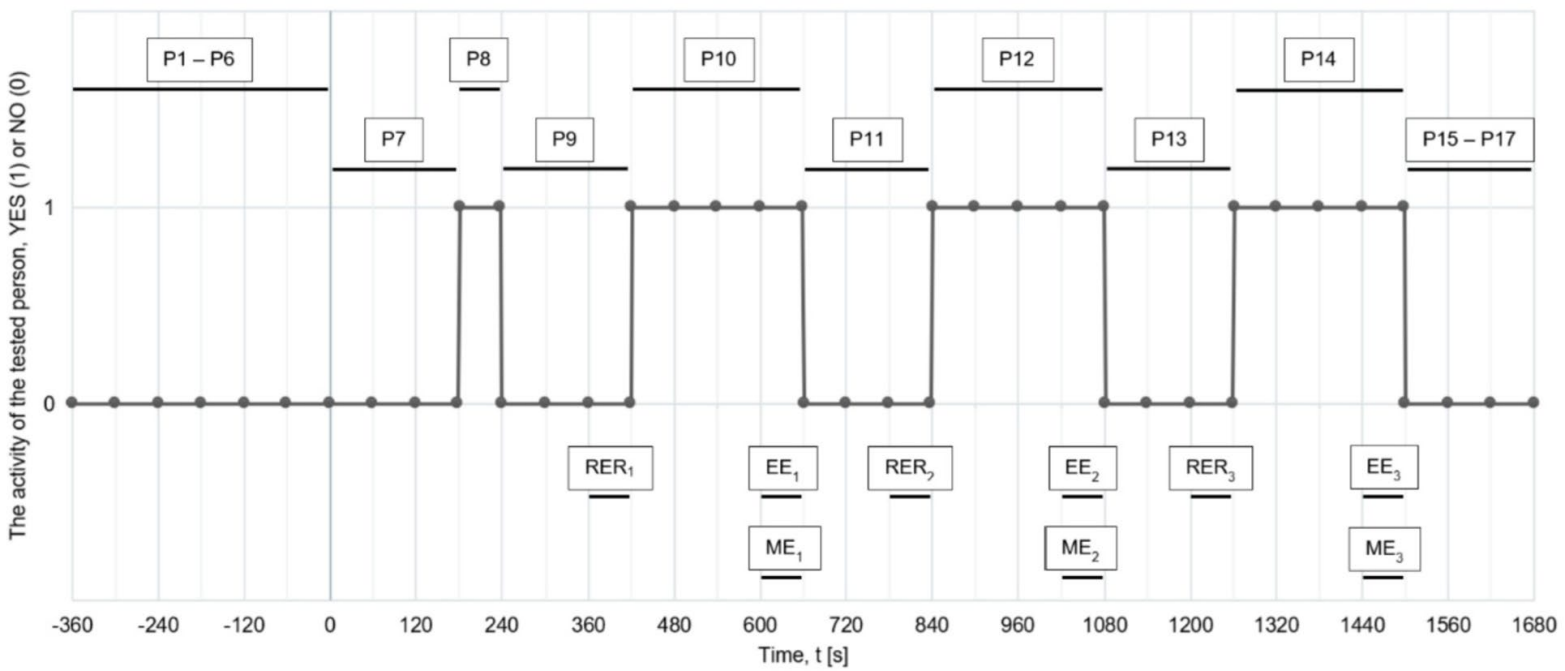
The test plan presented in the form of a graph.

The test procedure is illustrated in Fig. 9.

To determine gross and net efficiency, it was necessary to determine the energy expended by a person and the energy received on the power take-off shaft. The research defined the following areas for calculating efficiency: RER [J] – Resting Energy Rate, EE [J] – Energy Expenditure and ME [J] – Mechanical Energy. All defined research areas were determined for a time of 60 s and were located during the research according to the diagram in Fig. 9. A detailed definition of these energies was presented in a similar work on research on crankset^8^. Gross and net efficiency were calculated according to formula (1) and (2).

### Statistical analysis

In order to find the optimal angular position of the linear guides of the gantry drive, a statistical analysis was performed using Statistica v.13.1 software (Statsoft, PL). The results were described as mean values ​​and standard error. The Shapiro-Wilk test was used to assess the normality of data distribution. If the data did not have normal distribution, the Kruskal-Wallis ANOVA (KW), Dunn’s multiple comparisons test and the Mann-Whitney U (UMW) test were used to assess differences between groups in terms of individual parameters. A significance level of 0.05 was adopted to evaluate the hypotheses.

## Theory and calculations

The same formulas and calculation procedures were used as in the case of research on crankset^8^.

The formulas that were key to calculating gross and net efficiency are repeated below.

Gross efficiency:

1 $$\:{\eta\:}_{gross}=\frac{Mechanical\:Energy\:\left(ME\right)}{Energy\:Expenditure\:\left(EE\right)}*100\%$$

Net efficiency:

2 $$\:{\eta\:}_{net}=\frac{Mechanical\:Energy\:\left(ME\right)}{Energy\:Expenditure\:\left(EE\right)-Resting\:Energy\:Rate\:\left(RER\right)}*100\%$$

Mechanical Energy:

3 $$\:ME=\int\:M(\phi\:)\:d\phi\:$$

Below are the formulas that allow to calculate the exact position of the seat during tests for individual test subjects.

The exact position of the seat during the individual test – coordinate p:

4 $$\:p=P-m*\text{cos}\beta\:$$

The exact position of the seat during the individual test – coordinate r:

5 $$\:r=R+m*\text{sin}\beta\:$$

where P and R are constants resulting from the nominal position of the seat (see Fig. 8).

## Results

### Dynamic research

Data collected during dynamic tests were used to indicate what angular position of the linear guides maximizes the efficiency of the human-mechanism system. Ergonomic aspects were also assessed during dynamic tests. Table 1 describes the test sequence, the seat position selected by the subject and the preferred angle of inclination of the guides.

Efficiency calculations were performed using a partially automated program to reduce the amount of time spent on analyses. The analysis of the results indicating the best angle of inclination of the linear guides was subjected to statistical analysis described in the next subsection.

**Fig. 10:**
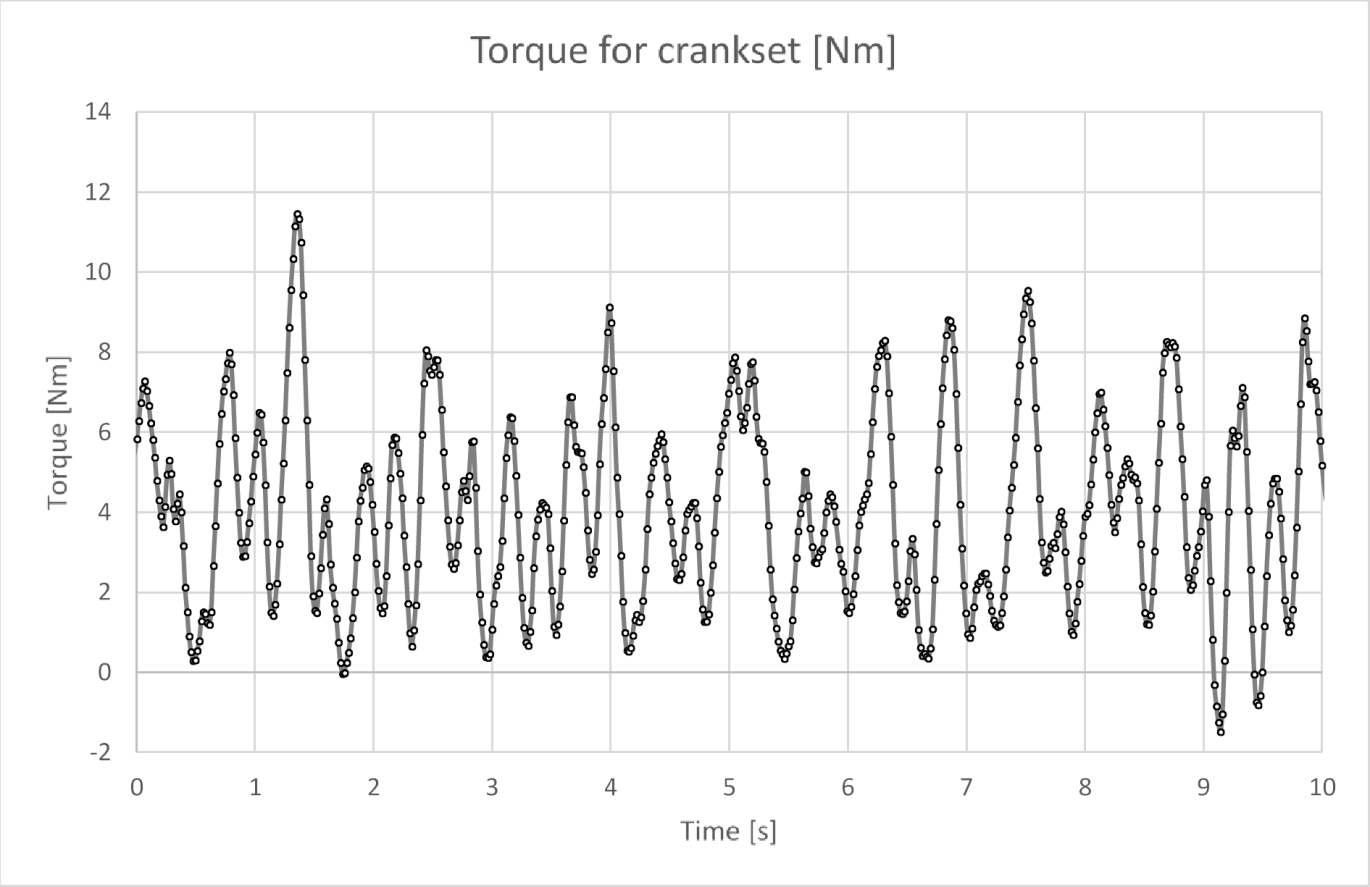
Diagram of M(t) for the crankset for 10 s of operation.

**Fig. 11:**
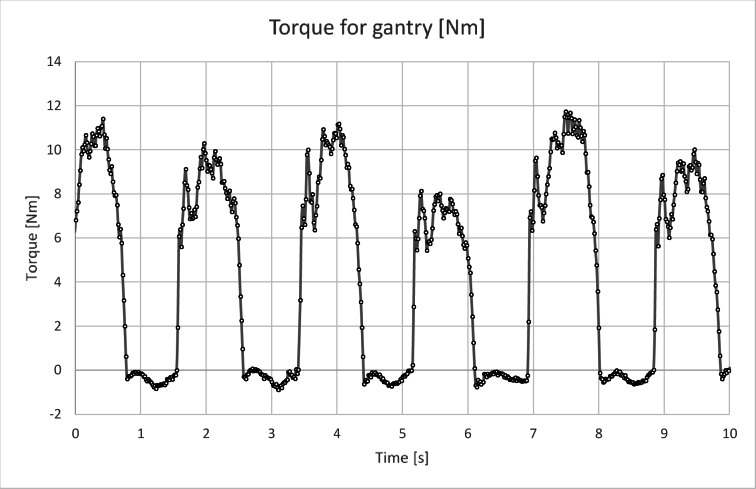
Diagram of M(t) for the gantry drive for 10 s of operation.

**Fig. 12:**
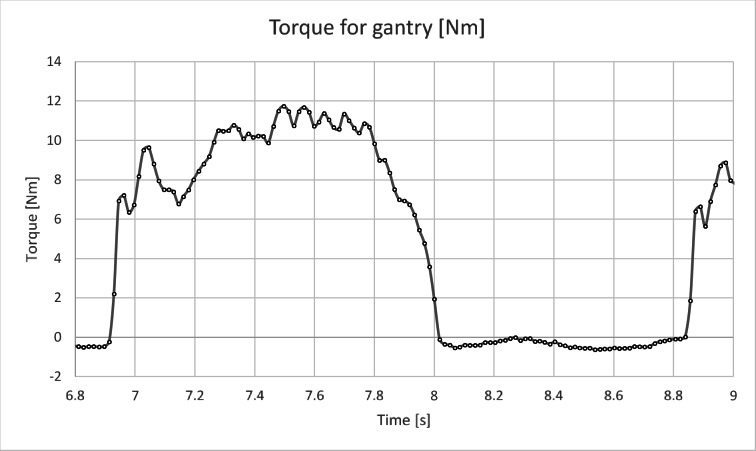
Diagram of M(t) for the gantry drive for one complete duty cycle.

Figure 10 shows the torque curve for the crankset over a period of 10 s, while Fig. 11 presents the torque curve for the gantry drive system over the same time period. As can be observed, both drives have completely different operating characteristics. The torque diagram for the gantry drive (Figs. 11 and 12) has a different character than expected at the level of theoretical considerations^4,29^. Theoretically, sharp cutoffs in the graph were expected, but studies have shown a slow increase in force and an even slower decline when the pressing phase ends and the pressure plate return phase in the trolley begins (see Fig. 11). The graph shows a peak in force occurring in 1/5 of the pressing cycle, then there is a decrease, then an increase in force to the value from the peak, which transforms into a plateau (see Fig. 12). The identical nature of the graph can be observed in all tested people.

The torque diagram for the crankset (Fig. 10) compared to the torque diagram for the gantry (Fig. 11) indicates a completely different level of muscle activity. It can be seen that the legs on the crankset are much more active, which, assuming a finite speed of the human muscles, leads to the conclusion that the gantry drive will allow for generating higher rotational speeds when the power limit will be removed.

Analysis of the recorded torque values shows that the torque peaks for the crankset often occur around 8 Nm (Fig. 10), while for the gantry drive around 10 Nm (Fig. 11). In the case of the gantry drive, the torque is generated by two legs, so the torque seen by one leg should be about 5 Nm. Based on this simple consideration, it can be concluded that the specific load (force) on the joints of the human legs in the case of the gantry drive should be lower than in the case of the crankset, assuming constant power generation.

The MA angular position was most frequently preferred by users, being selected as the best 16 times out of 36 tests. In turn, the TA angular position was selected 13 times out of 36 tests. The DA angular position was preferred least frequently, only 7 times out of 36 tests.

### Statistical analysis

**Fig. 13:**
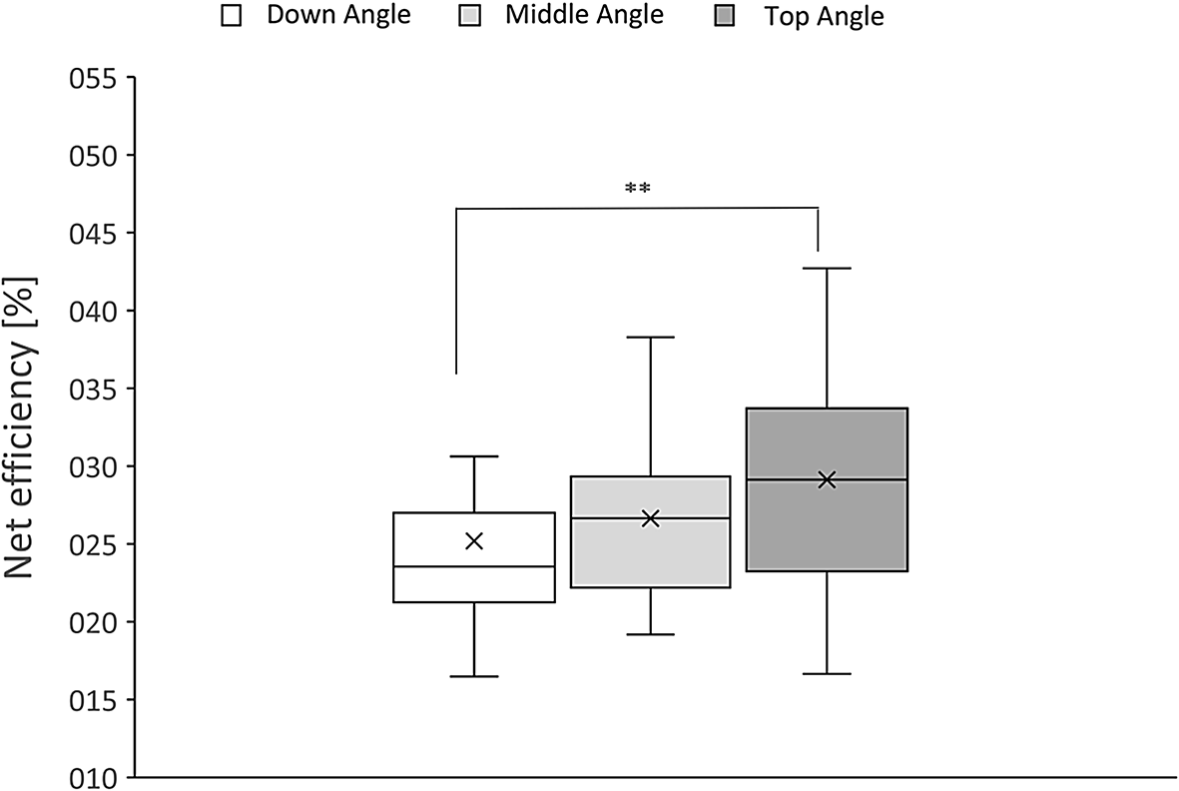
Net efficiency of different seat positions of gantry drive in 3 min observation (**p < 0.01, Dunn test – Top Angle vs. Down Angle).

Statistical analysis of the data obtained during the experiment showed differences in net efficiency (η_net_) between Down, Middle and Top Angle of the seat measured during whole 3 min of trial (H_(2,108)_ = 9.18; *p* < 0.01, Kruskal-Wallis ANOVA). They were also observed in shorter periods of time, both in the last 2 min (H_(2,108)_ = 9.91; *p* < 0.007, KW), and the last 1 min of observation within particular trial (H_(2,108)_ = 6.57; *p* < 0.04, KW). In the post-hoc examination greater efficiency was found for Top Angle (3 min: η_net_ = 29.13 ± 1.35%; 2 min: η_net_ = 28.16 ± 1.49%; 1 min: η_net_ = 26.74 ± 1.08%) in comparison to Down Angle (3 min: η_net_ = 25.19 ± 1.25%; *p* < 0.01, Dunn Test; *p* < 0.005, UMW test; 2 min: η_net_ = 23.72 ± 1.12%; *p* < 0.01, DT; *p* < 0.004, UMW; 1 min: η_net_ = 23.51 ± 1.13%; *p* < 0.05, DT; *p* < 0.01, UMW respectively), but not to Middle one (3 min: η_net_ = 26.65 ± 0.83%; *p* > 0.05 DT, UMW; 2 min: η_net_ = 24.98 ± 0.71%, *p* > 0.05, DT, UMW; 1 min: η_net_ = 24.12 ± 0.71%; *p* > 0.05 DT, UMW) (Fig. 13).

Regarding that in the detailed examination no statistical significance was shown between net efficiency for particular guide angle and different trial number (procedure points P10, P12, P14) during the experimental cycle (Down Angle: 3 min: H_(2,36)_ = 4.73; *p* > 0.1, KW; 2 min: H_(2,36)_ = 4.15; *p* > 0.1, KW; 1 min: H_(2,36)_ = 3.61; *p* > 0.2, KW; Middle Angle: 3 min: H_(2,36)_ = 5.16; *p* > 0.1, KW; 2 min: H_(2,36)_ = 4.51; *p* > 0.1, KW; 1 min: H_(2,36)_ = 4.24; *p* > 0.1, KW; Top Angle: 3 min: H_(2,36)_ = 2.06; *p* > 0.4, KW; 2 min: H_(2,36)_ = 1.48; *p* > 0.5, KW; 1 min: H_(2,36)_ = 0.22; *p* > 0.1, KW respectively), the trials were pooled for analysis up to 108.

**Fig. 14:**
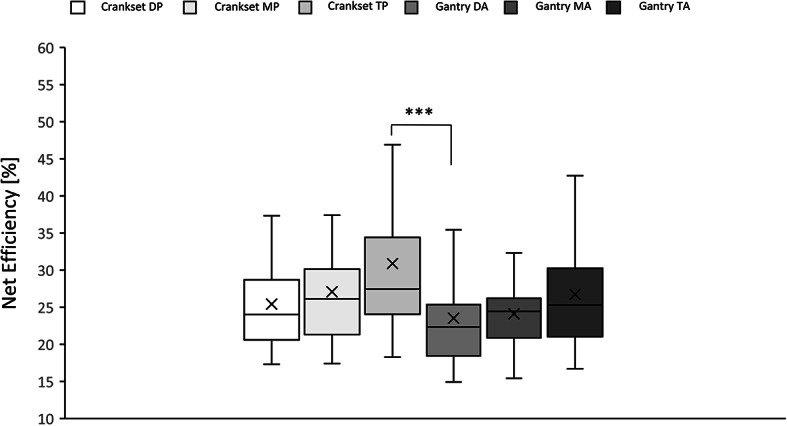
Net efficiency of different seat positions of the crankset and angle position of the gantry drive in the last minute of observation within a trial (***p < 0.001, Dunn test – TP vs. DA).

The analysis of net efficiency of the gantry drive when compared to the crankset one^8^ including various seat positions also showed some differences. They were noticed during the 3 min observation (H_(5,216)_ = 14.37; *p* < 0.01, Kruskal-Wallis ANOVA) as well as within shorter periods of time (H_(5,216)_ = 18.88; *p* < 0.01,KW – in the last 2 min of the trial and H_(5,216)_ = 19.67; *p* < 0.001,KW – in the last minute of the trial). Post-hoc testing revealed that in 3 min observation the gantry Down Angle (η_net_ = 25.19 ± 1.25%) had net efficiency lower that crankset both in the Middle (η_net_ = 29.81 ± 1.35%; *p* < 0.05, DT; *p* < 0.01, UMW) and Top Angle (η_net_ = 29.99 ± 1.65%; *p* < 0.05, DT; *p* < 0.01, UMW). Gantry Down Angle (η_net_ = 23.72 ± 1.12%) had also a smaller efficiency to these positions of crankset in last 2 min of trial (η_net_ = 28.44 ± 1.35%; *p* < 0.05, DT; *p* < 0.01, UMW and η_net_ = 29.44 ± 1.46%; *p* < 0.05, DT; *p* < 0.001, UMW, respectively). In the last minute gantry Down Angle (η_net_ = 23.51 ± 1.13%) showed lower efficiency only to crankset Top Position (η_net_ = 30.88 ± 1.85%; *p* < 0.001, DT; *p* < 0.0001, UMW) (Fig. 14).

**Fig. 15:**
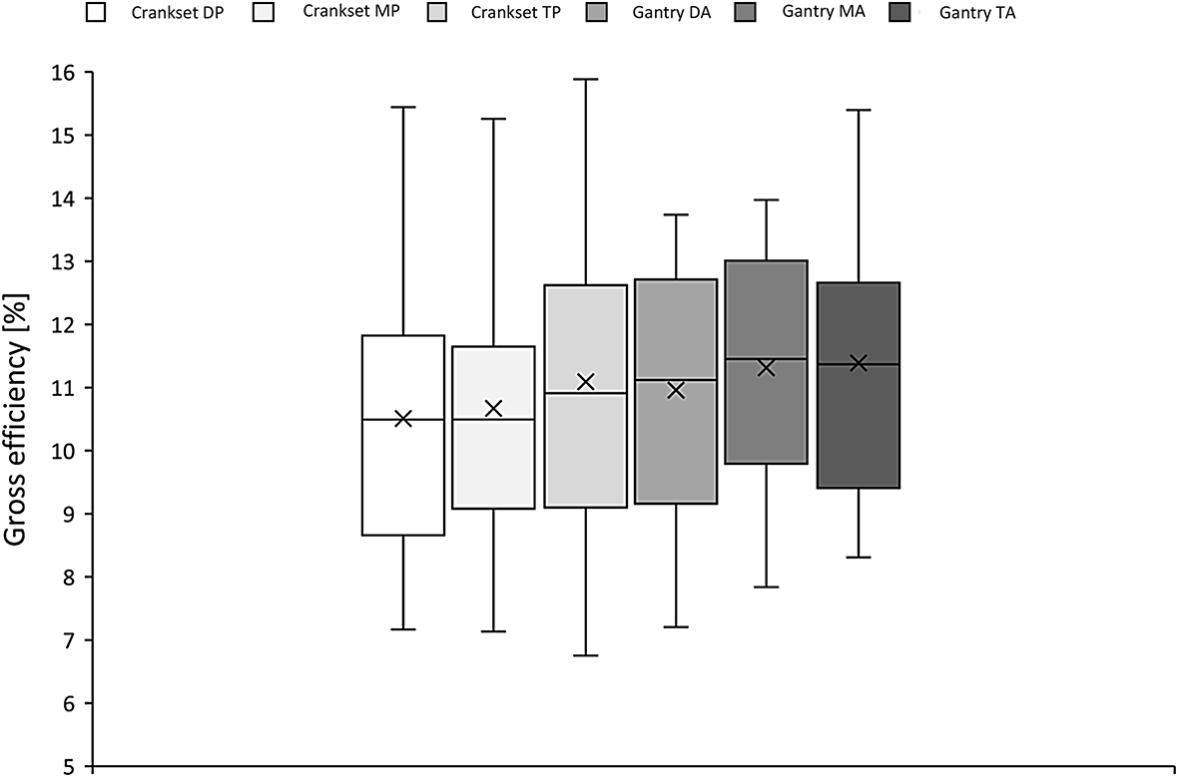
Gross efficiency of different seat positions of the crankset and angle position of the gantry drive in the last minute of observation within a trial (KW ANOVA – *p*>0.05).

Taking into account gross efficiency the KW ANOVA did not reveal any significant statistical differences between various positions of the seat in both in the crankset and gantry drive within any time period of observation in a trial (3 min: H_(5,216)_ = 7.05; *p* > 0.05, KW; 2 min: H_(5,216)_ = 6.92; *p* > 0.05, KW; 1 min: H_(5,216)_ = 5.9; *p* > 0.05, KW) (Fig. 15).

## Discussion

Comparative statistical analyses between the crankset and the gantry drive showed that crankset performed better when considering net efficiency (see Fig. 14). Comparative analysis for gross efficiency did not show any statistical differences (see Fig. 15). The braking torque in the case of the gantry drive was 1.2 Nm, and in the case of crankset it was ~ 0.8 Nm^8^, so it was relatively high, which was partly due to the unfavorable suspension of the gantry frame on the power take-off shaft. The load of the power take-off shaft by the gantry frame had an unfavorable effect, and the calculations were not corrected for these motion resistances. There are methods for realizing a more favorable attachment of the gantry drive to the frame. Considering the above, it seems safe to conclude that both techniques for receiving mechanical energy from a human at a load of 50 W are similar.

From a statistical point of view, the TA position is quite similar to the MA position (see Fig. 13). A similar relationship occurs in individual feelings collected from the respondents. This suggests that both angular positions are similar and the selection criterion may be different. In turn, the individual assessment of ergonomics of the angular position gave the result of 16/36 for MA, 13/36 for TA and 7/36 DA. It can be seen that TA and MA perform similarly in terms of both efficiency and individual assessment.

In a vehicle with an overall outline of 2.19 × 0.78 × 1.16 m (see Figs. 3 and 4) containing a gantry drive, it is difficult to accommodate the wheel drive unit with the gearbox, therefore the design approach in which the gantry is mounted in the vehicle in the TA position is advantageous because it allows for accommodating all the necessary drive elements, e.g. the gearbox in the front part of the vehicle under the gantry.

**Fig. 16:**
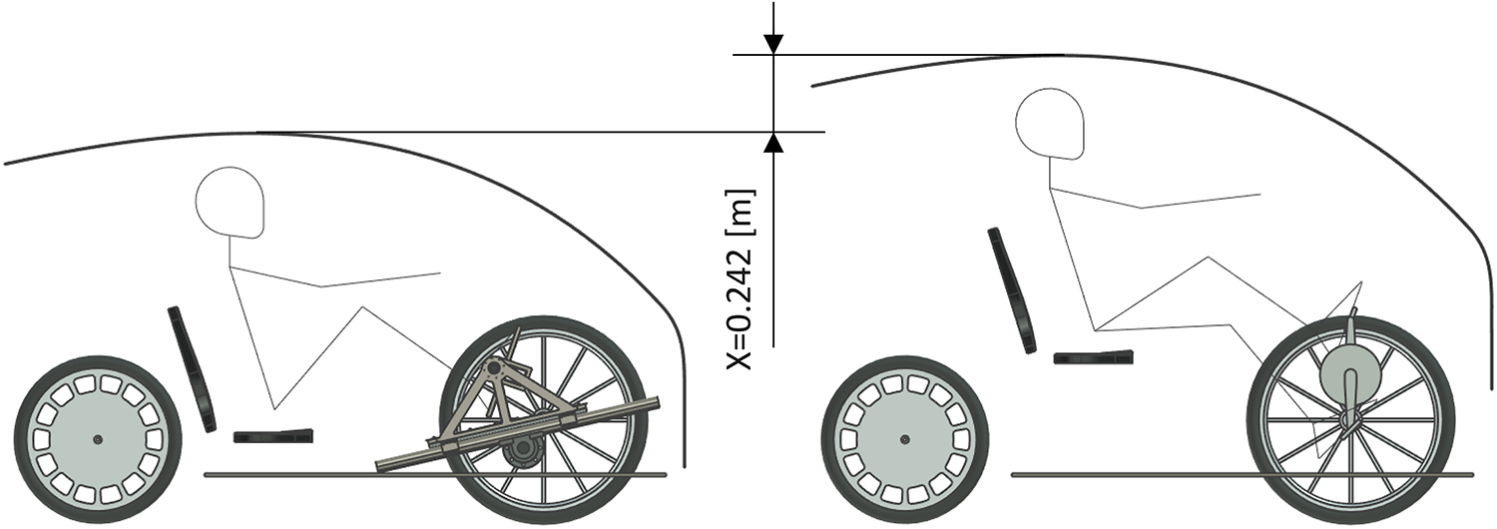
Difference in vehicle height between a personal vehicle equipped with the gantry drive and the crankset.

Below is a comparison of the gantry drive with crankset in terms of how these two mechanisms of receiving mechanical energy affect the structure of a road vehicle. Figure 16 shows the most favourable arrangement of the gantry drive and the most favourable position of the seat in relation to the crankset. The position of the foot on the pressure plate in the gantry drive was recreated based on a video recorded during the tests; the pressure plate is inclined by 58° relative to the linear guides of the gantry drive. The position of the feet on the pedals when crankset was also recreated from the video; the upper pedal is inclined 70° to the horizontal surface, while the lower pedal is inclined 40° to the horizontal surface. In the case of the overhead gantry, the human’s feet were placed essentially symmetrically on the pressure plate. In turn, in the case of crankset, the person pressed the pedals with their feet mainly in the central part of the foot. The analysis of the overall geometry of the vehicle was carried out for a person of 177 cm height – the proportions of individual body parts are consistent with the actual measurements of a single potential user of the vehicle. The position of the seat was m = 0.209 m. The distance from the top of the head to the roof was assumed to be 0.102 m, and the floor plate was at a height of 0.150 m from the road surface. In a vehicle equipped with crankset, the rotation axis of the mechanism is set at such a distance from the vehicle floor that a person does not hit the floor with his heel – the crankset axis is at a height of 0.307 m from the floor, and the cranks are 0.170 m long. It was assumed that the floor is 0.010 m thick. The vehicle can be 0.242 m lower when using the gantry drive, which reduces the frontal surface of the vehicle by approximately 19% and has a positive effect on aerodynamic resistance to movement. In the calculations, the vehicle width was assumed to be 0.780 m, and the vehicle height in the case of the gantry drive was 1.214 m, and the vehicle height in the case of the crankset was 1.456 m.

The gantry drive shown in Fig. 8 was tested. In this design, the trolley on which the pressure plate is installed is 0.43 m long, and the guides are 0.90 m long, so the trolley has a maximum stroke of 0.47 m. The tests have shown that if it is possible to adjust the position of the seat relative to the guide, the gantry stroke of 0.47 m is sufficient. It should be borne in mind that it is possible to significantly reduce the length of the trolley, e.g. to 0.10 m, and consequently it will be possible to shorten the guides and obtain additional space inside a personal vehicle.

The gantry drive aroused great interest among users and was enthusiastically received. Combining a gantry drive with a built-in three- or four-wheeled vehicle can significantly change the perception of drives powered by human muscles. Such a vehicle can be used in virtually any weather conditions, which should have a positive impact on people’s overall condition. People often give up cycling because they are afraid of rain or falling, especially when there is snow and frost. Many studies indicate that people who did not learn to ride a bicycle (two-wheeled bicycle with cranking) in childhood have very big problems learning it when they are adults^26,30–33^. A personal vehicle equipped with a gantry drive, legally qualified as a bicycle, can significantly positively change the perception of vehicles powered by human muscles. A passenger vehicle equipped with systems known from a passenger car, such as protection against weather conditions, full lighting, cooling and heating systems, may successfully compete directly with passenger cars, which are often used only by one person.

The analysis of the torque diagrams for the crankset and the overhead gantry shows a completely different nature of the operation of both mechanisms. With the gantry drive, the operation is much smoother, and leg movements occur at a much lower frequency, resulting in reduced inertial forces. The lower frequency of vibrations generated by the drive has a positive effect on the structures of both road and water vehicles by improving their stability. In the case of road vehicles, lower frequency should translate into a reduction in the number of cycles to which the structure will be subjected, at the cost of increased load per cycle of the mechanism. Lower frequency vibrations generated by the gantry compared to the crankset can be particularly beneficial in small boats, translating into a lower level of hull swings and thus causing less energy loss on movement.

The overhead gantry is an alternative to the sliding-seat rowing^34^ commonly used in boats. During operation of the gantry drive, the position of the legs changes, and the entire torso, head and arms remain motionless. In the case of a rowing drive, all parts of the body are accelerated and braked, which leads to the appearance of quite large inertia forces. Inertial forces in the case of paddle propulsion cause the nose of the watercraft to dive significantly. In the case of a gantry drive, this effect is minimized.

## Conclusions

Analyses and considerations of net and gross efficiency show that the gantry drive and crankset at a load of 50 W perform similarly.

The pilot studies carried out showed that the TA position has advantages over other positions tested. Future gantry drive studies will be carried out in road tests with the inclusion of ergonomic analysis in order to fully explore the area of ​​potential solutions in a real environment.

Despite their first contact with the gantry drive, the tested people used the gantry without difficulty and correctly. There was no difficulty in using it even though they had never tested it before. Moreover, the use of this drive in built-up vehicles can encourage people who are afraid to ride a typical bicycle to take up physical activity.

A vehicle equipped with a gantry drive can have a lower height while maintaining the spatial comfort of the vehicle user, which limits air resistance and reduces the weight of the vehicle casing.

The use of the gantry drive in typical 2-wheeled bicycles is difficult, because a person has 2 feet attached to the pressure plate, which makes it much more difficult to maintain balance when moving at low speeds. The gantry drive requires the installation of a 3rd wheel in the vehicle, which leads to an increase in the vehicle’s mass, and thus rolling resistance of the vehicle. Engineering analyses of the gantry drive have shown that it can have a similar degree of complexity compared to the commonly known crankset. There are solutions for the drive system for the gantry that have more elements than in the case of a drive with debarking. However, there are also configurations that are simpler than the crankset offers. The shaft axis from the crankset passes through the area where the cyclist’s leg works, which requires the use of a chain or other elements that allow the shaft axis receiving energy to be moved. The gantry drive allows the drive to be implemented directly to the drive wheel without the need for a drive chain, assuming that only 1 gear ratio is considered. Engineering analyses have shown that it is possible to implement the pressure plate guidance using various solutions such as the use of linear bearings^5^, guidance using a trolley with rollers^7^ and suspension of the pressure plate on a swinging lever^6^. Mounting the pressure plate on a swinging lever is one of the simplest solutions and has the highest degree of reliability, however, it requires a significant enlargement of the gantry mechanism.

The gantry drive is well suited for three-wheeled and four-wheeled vehicles, which, after being built and supported by an auxiliary engine/motor, can achieve the functional characteristics of a commonly known small passenger car. Due to its low level of complexity, such a means of transport can have a relatively low price and perform many of the tasks of a typical passenger car, especially over short distances. A personal vehicle can largely contribute to the development of cities, towns and villages. In cities, it can reduce traffic jams and accelerate the green transformation. In turn, in towns and villages it can significantly contribute to improving the lives of many people by increasing access to schools and work. Moreover, the vehicle can significantly solve the problems of social exclusion commonly observed in small towns due to the migration of people to larger towns.

### Limitations of the study

The experiment was developed as an initial project - a pilot study. Thus it is limited in many aspects – the load 50 W was accepted as safe for participants and it did not require specific laboratory background. The homogenous participant sample limited the number of variables that could affect the final result (e.g., gender, age, fitness level, overall health status etc.) and increased the power of statistical tests in subsequent analysis of the data. But obviously a future set of experiments using higher loads or variable load and more diverse participants is needed to generalize the results on the whole population. Such projects will be designed and implemented in future after completing phase 1 research. In addition, in the research we followed the path of the most homogeneous research group possible, because in the research we wanted to compare two different mechanisms (gantry drive and crankset). A homogeneous research group is a certain analogy to a stable power source, in the case of these studies, the human in the system being studied can be compared to a kind of engine/motor.

The conducted studies focused only on 3 selected positions of linear guides mounted in a specific way relative to the seat. It should be borne in mind that there may be other more favourable configurations, and also that there may be a more favourable trajectory of the pressure plate. This issue requires further research, especially in a real environment, because laboratory studies significantly limit the in-depth investigation of critical aspects of the drive.

It should be emphasized that the research was of a pilot nature, was conducted on a small group of young men and has a limited scope of application. The pilot nature dictated a population with little diversity to confirm that further research was worthwhile. The studies showed differences in the gantry and the crankset performance, so further research is worthwhile. Further research on the gantry drive is necessary on a larger, diverse population and preferably in a wide power spectrum to better understand the differences compared to the commonly known crankset. It is also important that the gantry drive can be significantly improved by reducing the resistance to movement, reducing the volume of the mechanism, and thus a significant reduction in the weight of the gantry is possible, which will certainly affect the final result, compared to the tested prototype. In addition, it is recommended to perform road tests, because laboratory tests have a number of limitations and do not provide a full picture of the issue being studied.

## Data Availability

Detailed data about the participants of the research are not publicly available due to the risk of identification. The raw baseline data collected during the tests and the calculated gross and net efficiencies are available from the corresponding author on reasonable request.
